# Longitudinal fatigue outcomes and prediction in inflammatory arthritis: a real-world digital cohort study

**DOI:** 10.1007/s00296-026-06308-9

**Published:** 2026-09-30

**Authors:** Dmytro Fedkov, Danylo Yevstifeiev, Oleg Iaremenko, Alexandra Dopfer-Jablonka, Uta Kiltz, Jacqueline Detert, Felix Lang, Abdullah Khalil, Peter Bramlage, Türker Kurt, Stefan Vordenbäumen

**Affiliations:** 1 Department of Internal Medicine with a course in Cardiology and Rheumatology, Bogomolets National Medical University, Kyiv, Ukraine; 2 Medical Center Medical Clinic Blagomed LLC, Kyiv, Ukraine; 3 Midaia GmbH, Heidelberg, Germany; 4 Bogomolets National Medical University, 13 Blvd Shevchenka, Kyiv, 01032 Ukraine; 5 Department for Rheumatology and Immunology, Hannover Medical School, Hannover, Germany; 6 Ruhr-Universität Bochum, Bochum, Germany; 7 Rheumazentrum Ruhrgebiet, Claudiusstrasse 45, 44649 Herne, Germany; 8 Institute for Pharmacology and Preventive Medicine, Cloppenburg, Germany; 9 Department of Rheumatology, St. Elisabeth-Hospital Meerbusch-Lank, Hauptstr. 74-76, 40668 Meerbusch, Germany; 10 Department of Rheumatology and Hiller Research Center, University Hospital Düsseldorf, Medical Faculty of Heinrich Heine University Düsseldorf, Düsseldorf, Germany

**Keywords:** Fatigue, Arthritis, Rheumatoid, Spondylarthritis, Psoriatic, Mobile applications, Self-management, Patient reported outcome measures, Longitudinal studies

## Abstract

Fatigue is a burdensome symptom in inflammatory arthritis (IA), yet meaningful longitudinal change remains poorly characterized in real-world settings. To characterize fatigue trajectories, quantify clinically meaningful improvement/worsening, and identify baseline predictors of fatigue outcomes in a digital IA cohort. We analyzed Midaia digital application data from patients with rheumatoid arthritis, psoriatic arthritis, axial spondyloarthritis, or mixed IA with baseline Brief Fatigue Inventory (BFI) data. Fatigue trajectories were modelled via cluster-robust longitudinal regression, with secondary landmark analyses classifying BFI improvement/worsening using a prespecified pragmatic 1.0-point BFI change threshold at weeks 12 (W12), 24 (W24), and 52 (W52). Predictors were examined using logistic regression, machine learning with area under the curve (AUC) and model interpretation, and inverse probability weighting (IPW) for loss to follow-up. The cohort (2,660 patients; 3,436 valid BFI observations; 485 with follow-up) was 78.3% female, mean age 45.6 ± 12.9 years, baseline BFI 5.2 ± 2.0. The unadjusted BFI slope was − 0.017 points/week (95% CI − 0.025 to − 0.008; *p* < 0.001; corresponding to − 0.20 points by W12, below the MCID); the IPW-adjusted slope was attenuated to − 0.003 points/week, suggesting that the crude estimate was sensitive to differential follow-up. At W12, 26.8% improved and 25.6% worsened, with similar proportions at W24 and W52. Higher baseline BFI predicted W12 improvement (OR 1.54) and lower worsening (OR 0.62); higher baseline Patient Global Assessment of Disease Activity predicted worsening (OR 1.02). Machine-learning discrimination was modest (AUC 0.651–0.720). Diet/nutrition engagement was associated with higher improvement (OR 1.78) and lower worsening (OR 0.45). Fatigue trajectories in IA were heterogeneous. Landmark improvement/worsening proportions were unchanged after IPW, whereas mean improvement was attenuated. Baseline fatigue severity dominated prediction; action-plan associations are hypothesis-generating.

**Supplementary Information:**

The online version contains supplementary material available at https://doi.org/10.1007/s00296-026-06308-9.

## Introduction

Fatigue is common across inflammatory rheumatic and musculoskeletal diseases, with reported prevalence estimates of approximately 40–80% in rheumatoid arthritis (RA) and affecting more than half of patients with axial spondyloarthritis (axSpA) and approximately half of patients with psoriatic arthritis (PsA), depending on the instrument and cut-off used [1–3]. It is consistently ranked by patients as one of the most disabling and impactful symptoms of inflammatory arthritis (IA) [4–6]. Despite its clinical importance, fatigue remains incompletely understood and difficult to manage in routine care: it is poorly predicted by inflammatory markers, responds inconsistently to disease-modifying antirheumatic drugs, and lacks robust biomarkers or validated models for predicting clinically meaningful change over time [7–10]. Accordingly, recommendations from the European Alliance of Associations for Rheumatology (EULAR) and the National Institute for Health and Care Excellence explicitly support assessment and management of fatigue as part of holistic rheumatology care [11–13].

The Brief Fatigue Inventory (BFI) is a validated nine-item patient-reported outcome instrument that assesses fatigue severity and fatigue-related interference, with item scores ranging from 0 to 10, where higher scores indicate greater fatigue burden [14]. While the BFI was originally developed and validated in cancer-related fatigue, subsequent evidence supports its use in rheumatic disease contexts. In patients with rheumatoid arthritis, the BFI demonstrated high consistency with the disease-specific Bristol Rheumatoid Arthritis Fatigue Multi-Dimensional Questionnaire (Kendall’s W = 0.759, *p* < 0.001; Pearson *r* = 0.669, *p* < 0.001) [15], and was validated across large rheumatoid arthritis cohorts (*N* = 7,760) alongside the Multi-dimensional Assessment of Fatigue and SF-36 vitality subscale, with equivalent sensitivity to change [16]. The selection of BFI in the present study was further informed by the digital platform context: instruments requiring more items impose substantially higher response burden in asynchronous digital formats, whereas the nine-item BFI with its 0–10 numerical response scale achieves high completion rates. A 1.0-point BFI change has been used as a pragmatic threshold for minimal clinically important difference (MCID). Because IA-specific and direction-specific BFI thresholds for improvement and worsening are not firmly established, alternative thresholds were examined in sensitivity analyses [17]. However, most evidence on changes in fatigue derives from controlled trial populations or selected cohorts, which may not fully reflect the heterogeneity, engagement patterns, and follow-up behavior of patients using digital health tools in routine care.

Digital health platforms offer an opportunity to collect longitudinal patient-reported outcome data at scale and to describe symptom trajectories in real-world populations [18]. The Mida Rheuma App (Midaia GmbH, Germany) is a CE-certified digital health application for adults with inflammatory arthritis, primarily from German-speaking countries, enabling self-reported tracking of symptoms, functional status, medication use, and engagement with structured self-management action plans [19, 20]. These action plans provide educational, motivational, and behavioral self-management support, but the application does not assign or modify antirheumatic medication. As in many digital cohorts, follow-up completion is heterogeneous; if patients with different baseline symptom burden are more or less likely to continue providing data, estimates of average change may be biased by differential loss to follow-up [21, 22].

The central clinical question was how fatigue changes over time in digitally followed patients with IA, how often clinically meaningful improvement or worsening occurs, and whether baseline patient characteristics or self-management engagement can help identify patients at risk of worsening fatigue. To address this question, the present study aimed to: (1) characterize longitudinal change in the BFI summary score over 56 weeks in a real-world digital IA cohort; (2) quantify the proportions of patients with clinically meaningful BFI improvement, stability, or worsening at W12, W24, and W52; (3) identify baseline predictors of W12 fatigue improvement and worsening using multivariable logistic regression and machine-learning approaches; (4) examine whether engagement with self-management action plan categories was associated with W12 fatigue outcomes; and (5) assess the robustness of findings to differential loss to follow-up using inverse probability weighting. The study was not designed to evaluate treatment effectiveness; rather, it describes individual fatigue trajectories and predictors of clinically meaningful change on the BFI.

## Methods

### Study design and participants

**Fig. 1:**
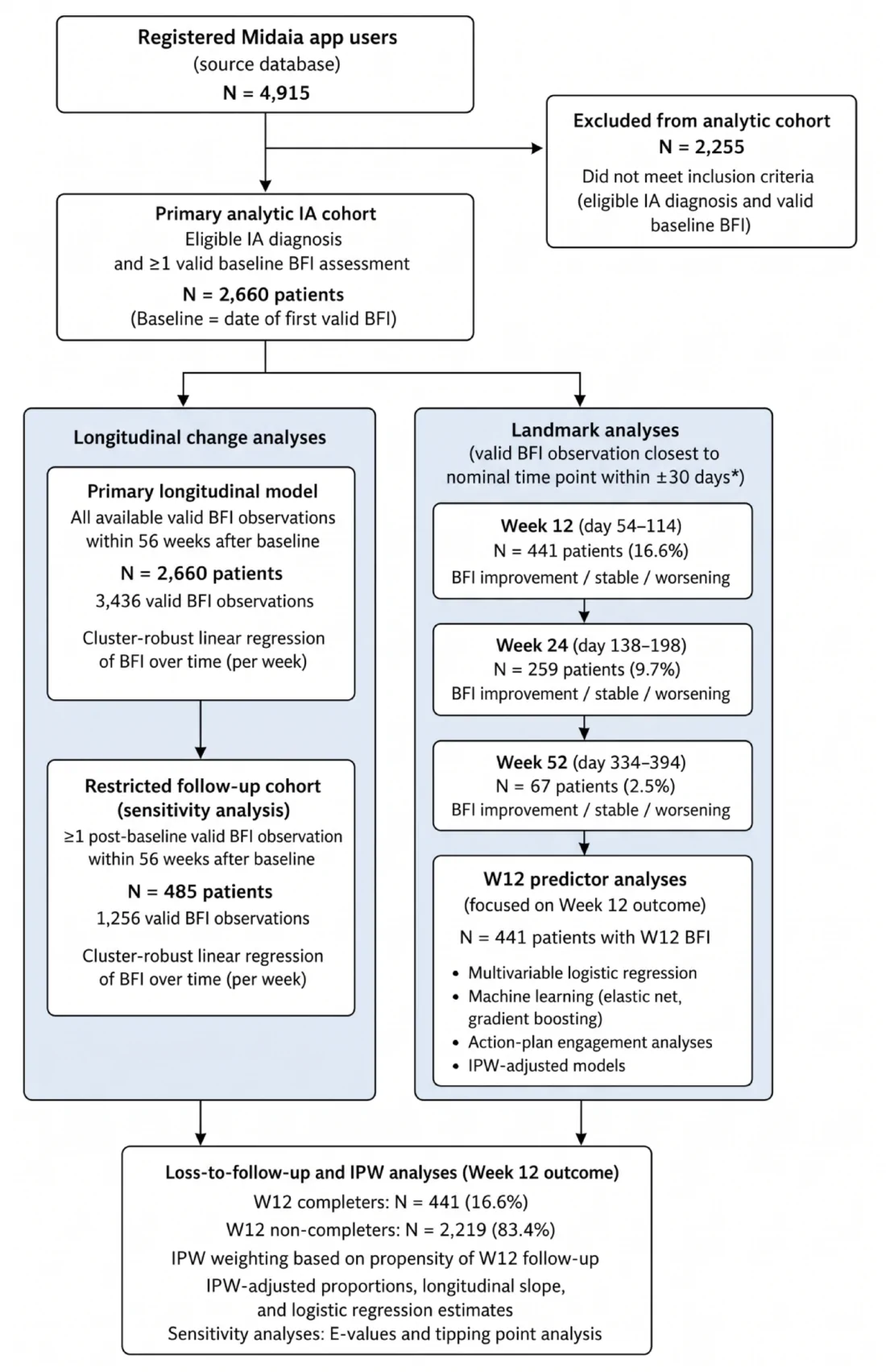
STROBE flow diagram of cohort selection and analytic samples. The diagram illustrates the derivation of the primary analytic cohort from all registered Midaia users, the follow-up cohorts used for longitudinal analyses, and the secondary landmark samples used for BFI improvement/stability/worsening analyses. The primary analytic cohort comprised 2660 patients with an eligible inflammatory arthritis diagnosis and at least one valid baseline Brief Fatigue Inventory (BFI) assessment. The primary longitudinal model included all available valid BFI observations obtained within 56 weeks after baseline (*N* =  2660 patients; 3436 observations). A restricted longitudinal sensitivity analysis was performed in a subset of patients with at least one post-baseline BFI observation (*N* = 485 patients; 1256 observations). W12, W24, and W52 cohorts represent secondary landmark analyses based on the availability of a BFI assessment near the respective follow-up timepoint. Loss-to-follow-up analyses compared W12 completers and non-completers and were used to derive inverse probability weighting (IPW) estimates. *Valid observation closest to the nominal time point (84, 168, 364 days from baseline) within the ± 30-day window. *BFI* Brief Fatigue Inventory, *IA* inflammatory arthritis, *IPW* inverse probability weighting, *ML* machine learning, *W12/W24/W52* week 12/week 24/week 52

This retrospective real-world cohort study used data from 4,915 registered users of the Mida Rheuma App (Midaia GmbH, Germany), a CE-certified medical device for adults with IA, collected between January 2022 and March 2026. Eligible IA diagnoses were based on the diagnosis category recorded by users at onboarding and included RA, PsA, axSpA, or mixed IA. The primary analytic cohort was selected from this source database using the following criteria: age ≥ 18 years, consent for research, use of app data, an eligible IA diagnosis, and at least one valid baseline BFI assessment. No a priori sample size calculation was performed; all eligible users who met the prespecified inclusion criteria during the study period were included. Baseline was defined as the date of the first valid BFI assessment. Users without an eligible baseline BFI or recorded eligible IA diagnosis were excluded and are shown together in Fig. 1. Reporting follows the STROBE recommendations for observational cohort studies. A completed STROBE checklist is provided as a supplementary file.

The present analysis draws on the same underlying Mida Rheuma App patient population as several prior reports from our group examining earlier, smaller subsets of this continuously growing dataset [19, 20, 22–24]; because the application’s user base and each patient’s available follow-up duration have expanded over time, the cohort analysed here is a larger and more extensively followed superset of those examined previously, rather than an independent sample.

### App assessments and follow-up structure

Assessments were completed by users in the application during routine app use. The application allowed repeated patient-reported outcome assessments over time, but no fixed study visit schedule was imposed. Therefore, follow-up time points were defined analytically. For W12, W24, and W52 analyses, the valid observation closest to the nominal time point (84, 168, and 364 days from baseline) within the prespecified ± 30-day window was used.

### Clinical variables and outcomes

Fatigue was assessed using the BFI summary score, with higher scores indicating greater overall fatigue burden [14, 15]. A valid BFI assessment was defined as an app-recorded BFI summary score generated from all nine completed BFI items. The primary outcome was longitudinal change in the BFI summary score over 56 weeks after baseline.

For secondary time-point analyses, clinically meaningful BFI change was classified at W12, W24, and W52. ΔBFI was calculated as follow-up BFI minus baseline BFI. BFI improvement was defined as ΔBFI ≤ − 1.0, stable fatigue as |ΔBFI| < 1.0, and BFI worsening as ΔBFI ≥ + 1.0. Sensitivity analyses used alternative MCID thresholds of ± 1.33 BFI points and paired thresholds of − 1.5 for improvement and + 1.7 for worsening [17, 25].

Baseline covariates included age, sex, body mass index (BMI), diagnosis category, pain score (PPAIN, 0-100 visual analogue scale) [26], Patient Global Assessment of Disease Activity (PtGADA, a 0–100 visual analogue scale reflecting the patient’s subjective perception of overall disease impact rather than objective inflammatory activity) [27], Routine Assessment of Patient Index Data 3 (RAPID3) [28], and Patient Health Questionnaire-4 (PHQ-4). The PHQ-4 was decomposed into a two-item depression subscale (anhedonia and hopelessness, items 1–2; range 0–6, score ≥ 3 indicating clinically elevated symptoms) and a two-item anxiety subscale (anxiety and worry, items 3–4; range 0–6, score ≥ 3 indicating clinically elevated symptoms) [29, 30].

Additional baseline characteristics evaluated descriptively included app-derived composite indices for sleep quality (scored from − 4 to + 4) and social support (scored from 0 to 20), utilizing the same operationalization as previously reported for this digital cohort [23]. Furthermore, self-reported baseline medication use (categorized into conventional synthetic DMARDs, biologic DMARDs, targeted synthetic DMARDs/JAK inhibitors, glucocorticoids, and NSAIDs) was extracted where voluntarily logged by users. Because medication tracking was an optional feature, these data were analyzed descriptively for the available subset rather than included in the primary multivariable predictive models.

### Action plan engagement

In addition to symptom and outcome tracking, the Mida Rheuma App provides structured self-management action plans. These action plans contain educational, motivational, and behavioral tasks intended to support disease self-management, but they do not replace medical care or involve prescribing or modifying medication. Each of the 43 action plan names was mapped to one of seven thematic categories: Diet & nutrition, Disease education, Exercise & movement, Fatigue management, Mindfulness & mental health, Pain management, and Sleep. Engagement with a category was defined as completion of at least one action plan task in that category before the W12 assessment window. Total task completion was calculated as the number of completed action plan tasks across all categories before W12 and was used in the exploratory dose-response analysis.

### Ethical considerations

The study was conducted in accordance with the ethical principles outlined in the Declaration of Helsinki (October 2024 revision) and the Good Clinical Practice guidelines. It was approved by the Local Ethics Committee of Medical Center Medical Clinic Blagomed LLC (Kyiv, Ukraine) (protocol #107/5, dated 13 August 2022). All patients provided informed consent before using the Mida Rheuma App.

### Statistical analysis

Descriptive statistics were used to summarize baseline characteristics and follow-up availability. Continuous variables are reported as mean and standard deviation or median and interquartile range, as appropriate. Categorical variables are reported as counts and percentages. Baseline correlations between continuous clinical and psychological variables were assessed using Spearman’s rank correlation coefficient (ρ). The distribution of baseline BFI scores across W12 fatigue outcome groups was visualized using raincloud plots to assess distributional overlap and potential regression-to-the-mean patterns, as the main predictor analysis was focused on W12 outcomes.

The primary analysis evaluated longitudinal changes in the BFI summary score. A linear regression model of BFI on time since baseline (in weeks) was fitted using all available valid BFI observations within 56 weeks of baseline. Cluster-robust standard errors at the patient level were used to account for within-individual correlation. The model estimated the average weekly change in BFI and the corresponding 95% confidence interval (CI).

Secondary landmark analyses described clinically meaningful changes in BFI summary score at W12, W24, and W52. At each landmark time point, patients were classified as having BFI improvement, stable fatigue, or BFI worsening according to the thresholds defined above. Proportions are reported with 95% CIs. Sensitivity analyses used alternative MCID thresholds to assess whether improvement and worsening proportions depended on the selected threshold. An exploratory W24 BFI improvement analysis using the same cross-validation pipeline was also performed and is reported in Supplementary Table S4.

Adjustment covariates were selected a priori based on their clinical relevance to fatigue outcomes and their availability within the app dataset. Multivariable logistic regression was used to identify baseline predictors of W12 fatigue improvement and W12 fatigue worsening. Two separate models were fitted: one with W12 BFI improvement as the dependent variable and one with W12 BFI worsening as the dependent variable. Independent variables were baseline BFI, PPAIN, PtGADA, PHQ-4, RAPID3, BMI, age, sex, and diagnosis category, with RA as the reference category. Results are reported as odds ratios (ORs), 95% CIs, and p values.

Associations between self-management action plan engagement and W12 fatigue outcomes were examined using exploratory logistic regression models. For each action plan category, crude and adjusted models were fitted for W12 BFI improvement and W12 BFI worsening. Adjusted models included baseline BFI, PPAIN, PtGADA, PHQ-4, RAPID3, age, and sex. In a dose-response sensitivity analysis, the total number of completed action plan tasks before W12 was modelled as a continuous predictor in an inverse-probability-weighted (IPW) logistic regression model.

Machine-learning analyses were performed as complementary predictive analyses to assess whether non-linear models provided additional discrimination beyond conventional regression and to explore patterns in non-linear predictors. Elastic net logistic regression (l1 ratio 0.5, C = 0.1) and gradient boosting (200 trees, learning rate 0.05) were evaluated using 5-fold stratified cross-validation for W12 BFI improvement and W12 BFI worsening. All preprocessing steps, including median imputation and feature standardization, were embedded within the cross-validation pipeline to prevent data leakage. Performance was assessed using the area under the receiver operating characteristic curve (AUC) and the area under the precision-recall curve, averaged across five held-out folds. Out-of-fold predicted probabilities were used to assess calibration. SHapley Additive exPlanations (SHAP) values were calculated for the gradient boosting model as a complementary interpretability analysis and were not used to designate the best-performing classifier.

Loss to follow-up was examined by comparing patients with and without W12 BFI follow-up using baseline characteristics and standardized mean differences (SMDs). A logistic regression model was fitted to estimate the probability of W12 follow-up completion based on measured baseline covariates. Stabilized inverse probability weights were calculated as $\:S{W}_{i}=P({R}_{i}=1)/P({R}_{i}=1\mid\:{X}_{i})$, where $\:{R}_{i}$ indicated W12 follow-up availability and $\:{X}_{i}$ denoted measured baseline covariates included in the completion model. Weights were trimmed at the 99th percentile. IPW-adjusted analyses included W12 improvement/worsening proportions, the longitudinal slope model, and logistic regression models for key predictors. To assess the sensitivity of the primary baseline-predictor associations to unmeasured confounding, E-values were calculated using the VanderWeele-Ding approach. Separately, a tipping-point analysis was performed to assess the sensitivity of the IPW-adjusted longitudinal slope to residual bias. Model discrimination for follow-up completion was evaluated using AUC.

Missing baseline data were handled according to the analytic model. Baseline BFI was complete by inclusion criterion. Age, sex, PPAIN, and PtGADA were complete, whereas BMI, RAPID3, and PHQ-4 had missing values and were imputed using the median in multivariable regression models. For standard logistic regression models, imputation statistics were derived from the corresponding analytic sample. For machine-learning models, median imputation was embedded within the cross-validation pipeline to prevent data leakage. Follow-up BFI values were not imputed; analyses at W12, W24, and W52 were restricted to patients with observed BFI data within the prespecified time window. All analyses were conducted using Python (v3.10) with the scikit-learn and statsmodels libraries. Two-sided p values < 0.05 were considered statistically significant.

## Results

### Cohort characteristics

Of 4915 registered app users in the source database, 2660 met the eligibility criteria for the primary analytic IA cohort (Fig. 1). The primary longitudinal analysis used 3,436 valid BFI observations from 2660 patients within 56 weeks after baseline, of whom 485 contributed more than one valid observation. Secondary landmark samples were smaller: 441 patients (16.6%) had a W12 BFI assessment, 259 (9.7%) had a W24 assessment, and 67 (2.5%) had a W52 assessment.

Baseline characteristics are presented in Table 1. The cohort comprised 2660 patients, of whom 2083 (78.3%) were female, with a mean age of 45.6 (SD 12.9) years and a mean BMI of 27.5 (SD 13.3). The most common diagnosis was rheumatoid arthritis (1432; 53.8%), followed by axial spondyloarthritis (644; 24.2%), psoriatic arthritis (370; 13.9%), and mixed inflammatory arthritis (214; 8.0%). Mean baseline BFI was 5.2 (SD 2.0), consistent with moderate-to-severe fatigue burden. Mean PtGADA was 58.5 (SD 25.4) and mean PPAIN was 53.8 (SD 25.5), reflecting substantial patient-perceived disease impact, and mean RAPID3 was 12.8 (SD 5.3).

**Table 1:** Baseline characteristics of the analytic cohort

Characteristic	Overall (*N* = 2660)
Age	45.6 (12.9)
Female, n (%)	2083 (78.3%)
BMI	27.5 (13.3)
RA, n (%)	1432 (53.8%)
PsA, n (%)	370 (13.9%)
axSpA, n (%)	644 (24.2%)
Mixed IA, n (%)	214 (8.0%)
BFI (0–10)	5.2 (2.0)
PPAIN (0–100)	53.8 (25.5)
PtGADA (0–100)	58.5 (25.4)
RAPID3 (0–30)	12.8 (5.3)
PHQ-4 (0–12)	4.2 (2.7)
PHQ-4 depression subscale (0–6)	2.30 (1.49)
PHQ-4 anxiety subscale (0–6)	1.92 (1.56)
Sleep Quality Score (− 4 – +4)	0.41 (1.72)
Social Support Score (0–20)	13.29 (4.28)

Values are mean (SD) unless otherwise stated

*axSpA* axial spondyloarthritis, *BFI* Brief Fatigue Inventory,* BMI* body mass index, *IA* inflammatory arthritis, *PHQ-4* Patient Health Questionnaire-4,* PPAIN* patient-reported pain score, *PsA* psoriatic arthritis, *PtGADA* Patient Global Assessment of Disease Activity, *RA* rheumatoid arthritis,* RAPID3* Routine Assessment of Patient Index Data 3, *SD* standard deviation

Regarding psychological and behavioral status, mean PHQ-4 total score was 4.2 (SD 2.7); subscale analysis revealed elevated depression symptoms (score ≥ 3) in 714 of 2065 patients with available data (34.6%, mean 2.30 ± 1.49) and elevated anxiety symptoms (score ≥ 3) in 600 (29.1%, mean 1.92 ± 1.56). Mean Sleep Quality Score was 0.41 (SD 1.72) and mean Social Support Score was 13.29 (SD 4.28). Variable-specific availability and handling of missing baseline covariates, including RAPID3 (22.7% missing) and PHQ-4 (22.5% missing), are reported in Supplementary Table S1. Baseline intercorrelations among clinical and psychological variables are shown in Figure S1.

Medication data, based on voluntary self-reported logging within the application, were available for 631 patients (23.7% of the cohort). Among patients with medication records, the most commonly reported drug classes were conventional synthetic DMARDs (csDMARDs; 276/631, 43.7%), bDMARDs (207/631, 32.8%), glucocorticoids (186/631, 29.5%), NSAIDs (159/631, 25.2%), and tsDMARDs/JAK inhibitors (83/631, 13.2%). Patients with recorded tsDMARD/JAKi use had significantly lower mean baseline BFI than those without recorded use (4.65 ± 1.91 vs. 5.31 ± 1.83; *p* = 0.003), while NSAID users had modestly higher baseline BFI (5.54 ± 1.87 vs. 5.11 ± 1.84; *p* = 0.008).

Patients with W12 BFI follow-up (completers, *N* = 441) differed systematically from those without it (Table S2). Completers had lower baseline BFI (4.8 vs. 5.3), lower pain (PPAIN 49.8 vs. 54.6), lower PtGADA (54.5 vs. 59.3), lower RAPID3 (11.9 vs. 13.0; *p* < 0.001) and lower PHQ-4 (3.8 vs. 4.3; *p* = 0.001); they also had modestly higher Sleep Quality Score (0.59 vs. 0.37) and Social Support Score (13.70 vs. 13.20), both *p* < 0.05, and were older (49.4 vs. 44.8 years, *p* < 0.001). Sex distribution did not differ significantly. Baseline covariate balance before and after IPW is shown in Figure S2.

### Longitudinal fatigue trajectories

**Fig. 2:**
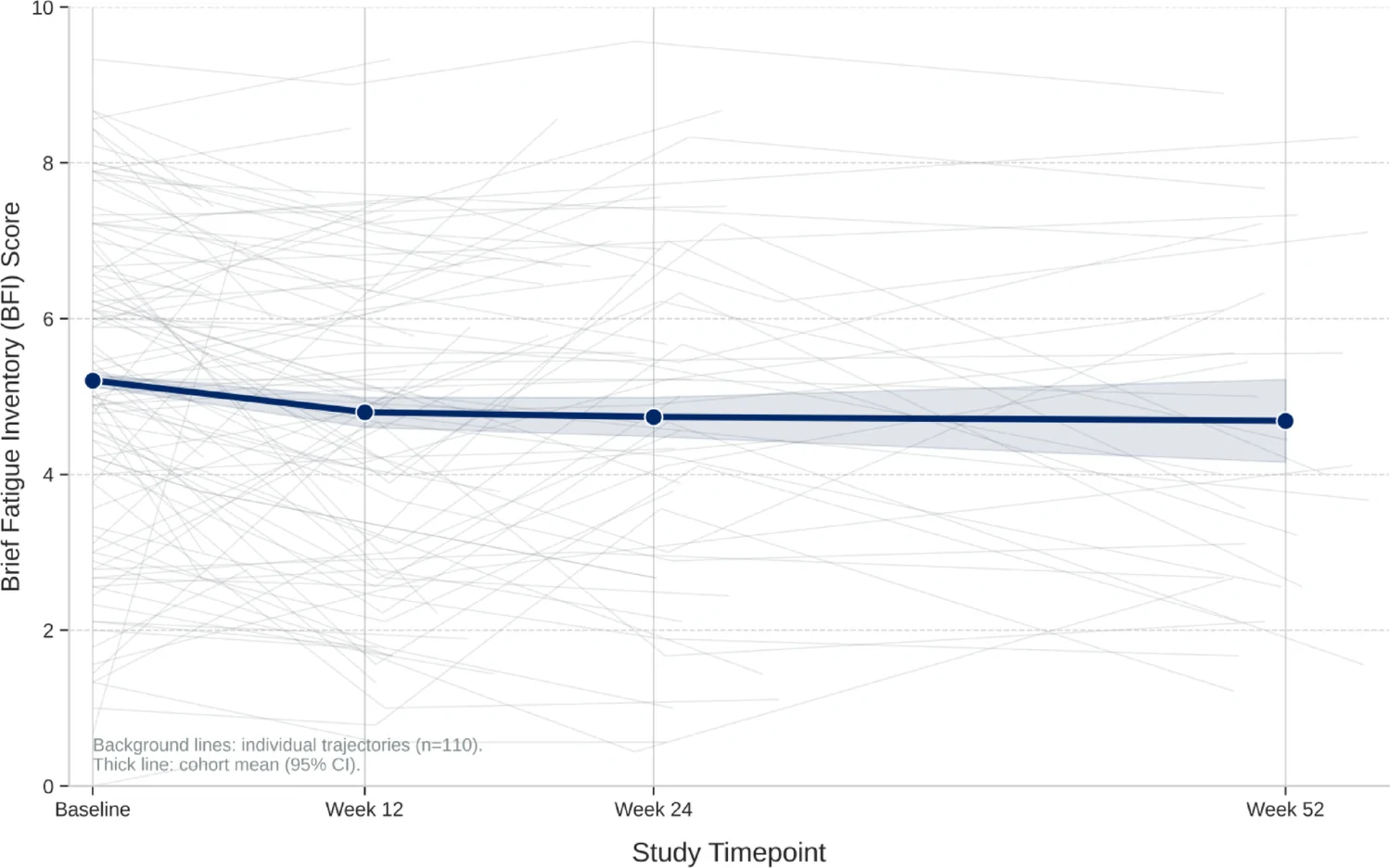
Longitudinal individual and mean trajectories of fatigue severity. Thin background lines represent a random sample of individual patient Brief Fatigue Inventory (BFI) trajectories across follow-up. The bold line represents the observed cohort mean BFI score at each assessment timepoint, with shaded areas indicating 95% CIs. Mean values are shown for baseline, week 12, week 24, and week 52 assessments. This visualization is descriptive and should be interpreted alongside the primary cluster-robust longitudinal model, which incorporated all available repeated BFI observations within the prespecified follow-up window. *BFI* Brief Fatigue Inventory, *CI* confidence interval

In the primary longitudinal model (*N* = 2660 patients; 3,436 valid BFI observations), the unadjusted BFI slope was − 0.017 points per week (95% CI − 0.025 to − 0.008; *p* < 0.001) (Table 2). This translates to an estimated group-average improvement of − 0.20 BFI points at W12, well below the MCID of 1.0. Individual observed BFI changes varied widely, ranging from clinically meaningful improvement to clinically meaningful worsening (Fig. 2). In the restricted follow-up cohort (*N* = 485 patients with at least one post-baseline BFI observation; 1256 valid BFI observations), the BFI slope was − 0.005 points per week (95% CI − 0.013 to + 0.003; *p* = 0.23).

**Table 2:** Cluster-robust linear regression of BFI over time

Parameter	Estimate	95% CI	*p* value
Model 1: Full cohort (*N* = 2660 patients; 3436 BFI observations)			
Intercept	5.17	5.10–5.25	< 0.001
Slope (per week)	−0.017	−0.025 to − 0.008	< 0.001
Implied change at W12	−0.20	−0.30 to − 0.10	−
Implied change at W24	−0.40	−0.59 to − 0.20	−
Implied change at W52	−0.86	−1.28 to − 0.44	−
Model 2: Restricted follow-up cohort (*N* = 485 patients; 1256 BFI observations)			
Slope (per week)	−0.005	−0.013 to + 0.003	0.23
Implied change at W12	−0.06	−0.15 to + 0.04	−
Implied change at W52	−0.25	−0.66 to + 0.16	−

The model was fitted using all available valid BFI observations within 56 weeks after baseline. Standard errors were clustered at the patient level. Time was modelled in weeks since baseline. Negative estimates indicate lower fatigue over time. Implied changes are calculated from the per-week slope

*BFI* Brief Fatigue Inventory, *CI* confidence interval, *W12/W24/W52* week 12/week 24/week 52

### Fatigue improvement and worsening analysis

**Fig. 3:**
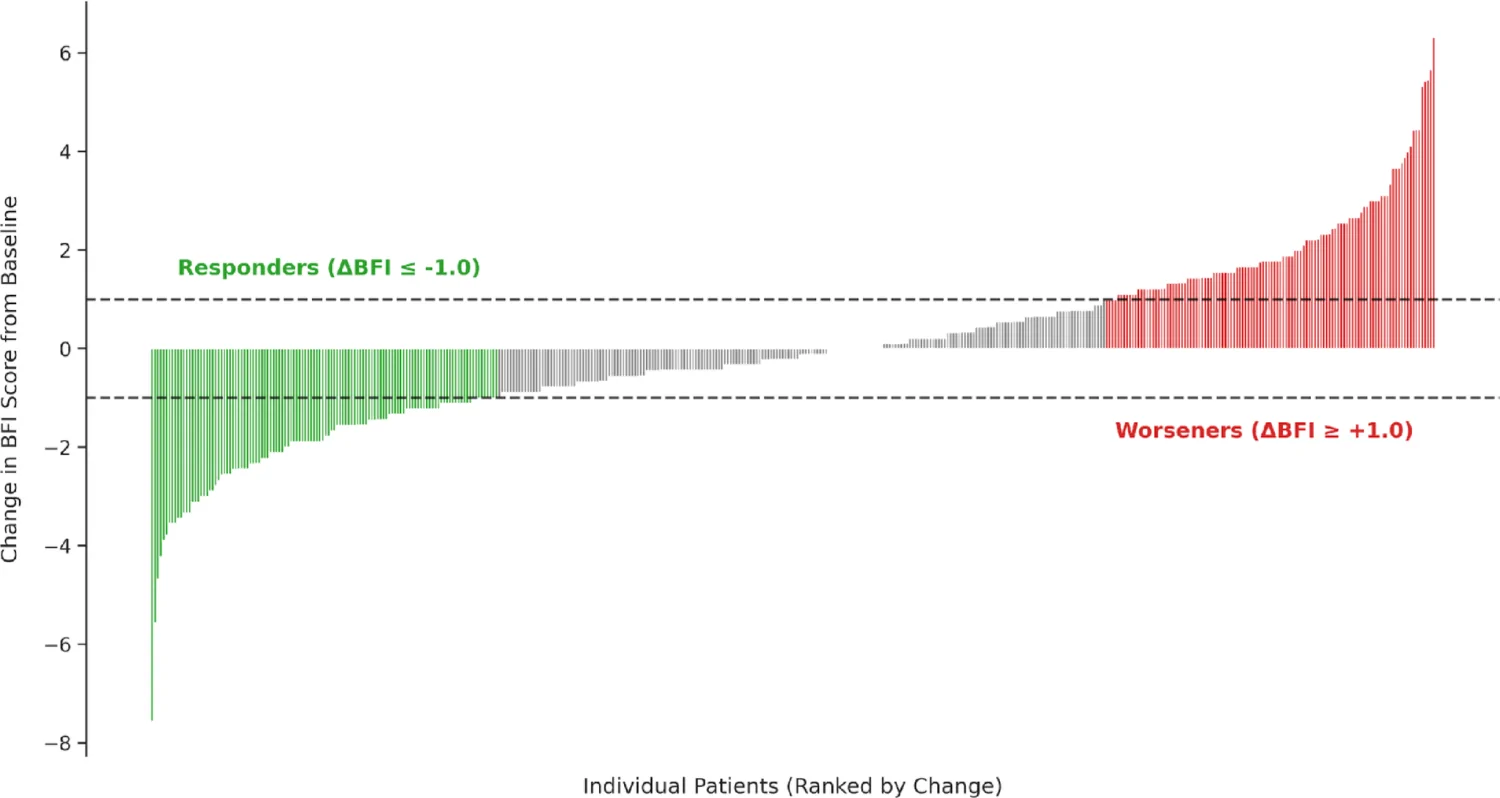
Waterfall plot of individual BFI changes from baseline to week 12. Each bar represents one patient with W12 landmark BFI and is ranked by ΔBFI. ΔBFI was calculated as W12 BFI minus baseline BFI; negative values indicate improvement and positive values indicate worsening. Dashed lines indicate the primary MCID threshold of ± 1.0 BFI point. *BFI* Brief Fatigue Inventory, *MCID* minimal clinically important difference, *W12* week 12

In secondary landmark analyses, despite only modest average change in the primary longitudinal model, observed individual BFI changes were heterogeneous (Table 3; Fig. 3). At W12, 26.8% of patients had clinically meaningful BFI improvement, and 25.6% had clinically meaningful BFI worsening. Improvement and worsening proportions were similar across timepoints (W24: 28.6% and 26.6%; W52: 32.8% and 25.4%). Approximately 42–48% of patients remained stable at each time point. These proportions were consistent across MCID sensitivity thresholds (Table S3).

**Table 3:** Clinically meaningful BFI improvement and worsening at weeks 12, 24, and 52

Timepoint	*N*	BFI improvement *n* (%)	Stable *n* (%)	BFI worsening *n* (%)	Mean ΔBFI (SD)
W12	441	118 (26.8%)	210 (47.6%)	113 (25.6%)	0.0 (1.8)
W24	259	74 (28.6%)	116 (44.8%)	69 (26.6%)	0.0 (1.8)
W52	67	22 (32.8%)	28 (41.8%)	17 (25.4%)	− 0.2 (1.7)

Secondary landmark analyses used the valid BFI observation closest to the nominal time point within the prespecified ± 30-day window. ΔBFI was calculated as follow-up BFI minus baseline BFI; negative values indicate improvement. Clinically meaningful BFI improvement was defined as ΔBFI ≤ − 1.0, stable fatigue as |ΔBFI| < 1.0, and clinically meaningful BFI worsening as ΔBFI ≥ + 1.0

*BFI* Brief Fatigue Inventory, *MCID* minimal clinically important difference, *SD* standard deviation, *W12/W24/W52* week 12/week 24/week 52

### Predictors of W12 BFI improvement and worsening

In multivariable logistic regression for W12 BFI outcomes (*N* = 441), higher baseline BFI was the strongest predictor of clinically meaningful W12 BFI improvement (OR 1.54, 95% CI 1.31–1.81; *p* < 0.001) and was inversely associated with W12 BFI worsening (OR 0.62, 95% CI 0.53–0.72; *p* < 0.001) (Table 4, Fig. S3).

**Table 4:** Multivariable logistic regression for W12 BFI improvement and worsening

Predictor	OR (BFI improvement)	95% CI	*p*	OR (BFI worsening)	95% CI	*p*
BFI baseline	**1.54**	**1.31–1.81**	< 0.001	**0.62**	**0.53–0.72**	< 0.001
PPAIN baseline	1.00	0.99–1.02	0.848	0.99	0.98–1.01	0.558
PtGADA baseline	1.00	0.98–1.01	0.679	**1.02**	**1.00–1.04**	0.034
RAPID3 baseline	0.93	0.86–1.00	0.060	1.00	0.93–1.08	0.958
PHQ-4 baseline	0.96	0.86–1.08	0.495	0.92	0.81–1.05	0.233
BMI baseline	1.01	0.97–1.05	0.666	1.03	0.98–1.07	0.224
Age (years)	1.02	1.00–1.04	0.093	0.99	0.97–1.01	0.148
Female sex	1.24	0.69–2.24	0.469	1.52	0.81–2.84	0.192
PsA vs. RA	0.97	0.50–1.87	0.919	1.31	0.65–2.66	0.447
axSpA vs. RA	0.99	0.58–1.71	0.979	0.83	0.46–1.49	0.529
Mixed vs. RA	0.95	0.40–2.25	0.900	0.84	0.33–2.14	0.716

Odds ratios are reported per one-unit increase unless otherwise stated. PPAIN and PtGADA are 0-100 visual analogue scales. RA was the reference diagnosis category. W12 BFI improvement was defined as ΔBFI ≤ − 1.0 and W12 BFI worsening as ΔBFI ≥ + 1.0. Bold indicates statistically significant associations (p < 0.05).

*axSpA* axial spondyloarthritis, *BFI* Brief Fatigue Inventory, *BMI* body mass index, *CI* confidence interval, *OR* odds ratio, *PHQ-4* Patient Health Questionnaire-4, *PPAIN* patient-reported pain score, *PsA* psoriatic arthritis, *PtGADA*, Patient Global Assessment of Disease Activity, *RA* rheumatoid arthritis, *RAPID3* Routine Assessment of Patient Index Data 3, *W12* week 12

Higher baseline PtGADA was independently associated with worsening W12 BFI (OR 1.02, 95% CI 1.00–1.04; *p* = 0.034). No other baseline predictor reached conventional statistical significance at W12. Predictor analyses focused on W12 because it was the predefined main time point for clinically meaningful BFI improvement and worsening and had the largest follow-up sample size.

### Machine-learning prediction of W12 BFI outcomes

Machine-learning models showed modest discrimination for W12 BFI outcomes. Elastic net logistic regression outperformed gradient boosting for W12 BFI improvement (AUC 0.651 vs. 0.603) and W12 BFI worsening (AUC 0.720 vs. 0.680). At W24, gradient boosting slightly outperformed elastic net for BFI improvement (AUC 0.711 vs. 0.669) (Table S4). ROC curves and calibration plots are shown in Figure S4. Although the elastic net showed the highest W12 cross-validated AUC, the gradient-boosting SHAP analysis was used as a complementary non-linear interpretability analysis and confirmed that baseline BFI was the dominant driver of predicted W12 BFI improvement (Figure S5).

### Self-management action plan engagement

**Fig. 4:**
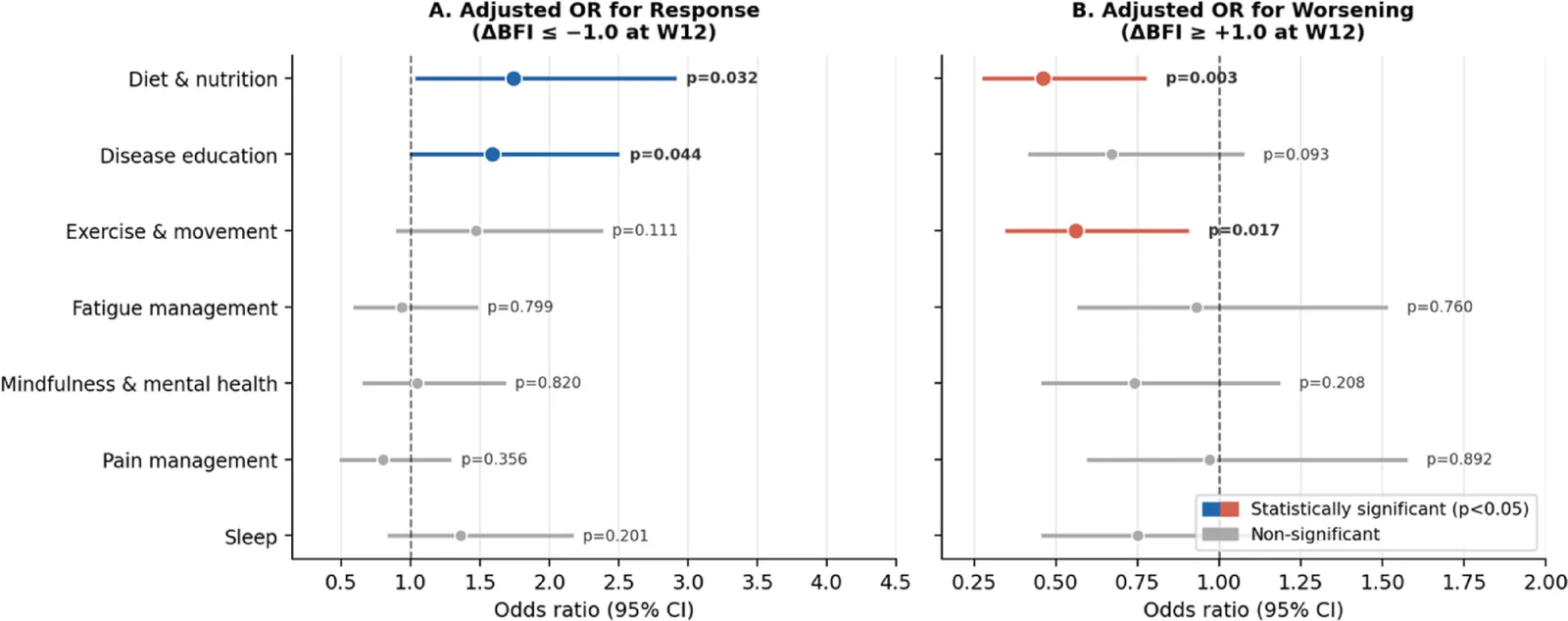
Adjusted associations between self-management action plan engagement and W12 BFI outcomes. Adjusted odds ratios are shown for W12 BFI improvement (Panel A) and W12 BFI worsening (Panel B) by action plan category. Models were adjusted for baseline BFI, PPAIN, PtGADA, PHQ-4, RAPID3, age, and sex. These analyses are exploratory and observational. Error bars represent 95% CIs. *BFI*, Brief Fatigue Inventory, *CI* confidence interval, *OR* odds ratio, *PHQ-4* Patient Health Questionnaire-4, *PPAIN* patient-reported pain score, *PtGADA* Patient Global Assessment of Disease Activity, *RAPID3* Routine Assessment of Patient Index Data 3, *W12* week 12

Among measured potentially modifiable variables, engagement with selected self-management action plan categories showed exploratory associations with W12 BFI outcomes (Table S5). In adjusted logistic regression using the primary ± 1.0 BFI threshold, Diet & nutrition engagement was associated with higher odds of W12 BFI improvement (OR 1.78, 95% CI 1.07–2.96; *p* = 0.026) and lower odds of W12 BFI worsening (OR 0.45, 95% CI 0.27–0.75; *p* = 0.002). Disease education engagement was associated with higher odds of W12 BFI improvement (OR 1.91, 95% CI 1.16–3.16; *p* = 0.011), whereas its association with W12 BFI worsening did not reach conventional statistical significance (OR 0.63, 95% CI 0.39–1.02; *p* = 0.063). Exercise & movement engagement was not significantly associated with W12 BFI improvement or worsening after adjustment. In the dose-response analysis, each additional 10 completed tasks was associated with lower odds of W12 BFI worsening (OR 0.95, 95% CI 0.92–1.00; *p* = 0.030), whereas the association with W12 BFI improvement was not statistically significant. Sensitivity analyses using alternative MCID thresholds showed generally consistent directionality for Diet & nutrition and task completion, but some action-plan associations were threshold-dependent and should therefore be interpreted as exploratory (Fig. 4).

### Loss to follow-up and IPW

The propensity model for W12 follow-up completion achieved an AUC of 0.624. Older age (OR 1.03/year; *p* < 0.001) and male sex (OR 1.37; *p* = 0.021) were the strongest predictors of continued follow-up (Table S6).

IPW-adjusted W12 improvement and worsening proportions were nearly identical to the unweighted estimates: BFI improvement was 27.2% after IPW versus 26.8% unweighted, and BFI worsening was 24.0% after IPW versus 25.6% unweighted.

In contrast, the IPW longitudinal slope attenuated substantially to − 0.003 BFI points per week (95% CI − 0.011 to + 0.005; *p* = 0.43), compared with the unweighted slope of − 0.017 (*p* < 0.001).

In IPW logistic regression, baseline BFI remained associated with W12 BFI improvement (OR 1.49; *p* < 0.001) and was inversely associated with W12 BFI worsening (OR 0.63; *p* < 0.001). The association between PtGADA and W12 BFI worsening attenuated under IPW (*p* = 0.085) but remained directionally consistent.

Sensitivity analyses for unmeasured confounding supported the robustness of the primary BFI associations. The E-value for the BFI-improvement association (OR 1.54) was 2.45 (95% CI bound: 1.95), indicating that an unmeasured confounder would need to be associated with both baseline BFI and W12 BFI improvement by a risk ratio of at least 2.45-fold to fully explain the observed association. The corresponding E-value for the inverse BFI-worsening association (OR 0.62) was 2.61 (CI bound: 2.12). The tipping point analysis for the IPW longitudinal slope showed that residual unmeasured confounding would need to bias the slope by more than 0.011 BFI points per week, equivalent to 0.57 BFI points over 52 weeks, to render it statistically significant.

## Discussion

The main finding of this study is that fatigue in IA followed heterogeneous trajectories in a real-world digital cohort. Although the average longitudinal change in BFI was small, secondary time-point analyses showed that approximately one quarter of observed patients improved, approximately one quarter worsened, and nearly half remained stable at each assessed time point. This distinction is clinically important because population-average improvement may obscure substantial individual deterioration. These findings support individual-level fatigue monitoring rather than relying on average group change alone [31, 32].

Baseline BFI was the dominant predictor of both W12 BFI improvement and W12 BFI worsening. This pattern is consistent with regression to the mean: patients with higher baseline fatigue had greater room for improvement, whereas patients with lower baseline fatigue were more likely to meet the worsening threshold during follow-up [24, 33]. This pattern underscores that baseline severity mechanically influences threshold-defined change; consequently, the strong association of baseline BFI with subsequent improvement or worsening should not be interpreted causally. The association between higher PtGADA and worsening of W12 BFI suggests that patient-perceived global disease burden may help identify patients at risk of fatigue deterioration, consistent with prior evidence that fatigue is more closely related to patient-reported disease impact than to objective inflammation alone [25, 33–36].

The self-management action plan analysis provides the most clinically modifiable signal in this study, although it remains exploratory. Engagement with selected action plan categories, particularly Diet & nutrition and Disease education, was associated with more favourable W12 BFI outcomes after adjustment for baseline symptom burden (Table S5). Diet & nutrition showed the most consistent association, with higher odds of W12 BFI improvement and lower odds of W12 BFI worsening. Disease education was associated with higher odds of W12 BFI improvement, while higher total task completion was associated with lower odds of W12 BFI worsening. Associations for other action-plan categories were weaker or threshold-dependent. These findings suggest that structured digital self-management content may help identify behavioural engagement patterns linked to more favourable fatigue trajectories, but they do not establish treatment efficacy or causality.

Several mechanisms may plausibly explain these associations. Patients who engage with education, nutrition, or exercise-related tasks may also have greater self-management capacity, motivation, or support, which could influence fatigue outcomes independently of the specific content. Reverse causation is also possible, as patients whose fatigue is already improving may be more likely to continue engaging with the app. Nevertheless, the consistency of the direction of associations and the dose-response pattern, in which higher total task completion was associated with reduced odds of W12 BFI worsening, support further prospective evaluation of targeted digital self-management strategies in IA [37–39].

Regarding loss to follow-up, patients who continued to provide W12 BFI data were systematically healthier at baseline, indicating differential dropout from follow-up. The modest discrimination of the completion model further suggests that measured baseline characteristics only partly explained follow-up completion, leaving room for unmeasured behavioral or contextual factors. The observed group-level improvement in fatigue should therefore be interpreted with caution, as it may be partly explained by selective retention of lower-burden patients. Importantly, adjustment for loss to follow-up did not materially affect the proportions of patients with clinically meaningful BFI improvement or worsening. This distinction is clinically relevant: average change estimates in digital cohorts may be sensitive to selective follow-up, whereas the observed distribution of individual-level improvement and worsening appeared more robust in this dataset [40, 41].

The complementary machine-learning analyses provided only modest discrimination for W12 BFI improvement and worsening, broadly consistent with previous prediction work in IA, where reported AUC values often fall within the modest range of approximately 0.60–0.75 [31, 42, 43]. These findings suggest that baseline variables alone have limited predictive power for future BFI improvement or worsening. This is clinically plausible, as fatigue is multidimensional and dynamic, and relevant determinants such as sleep, coping, physical activity, and fluctuating disease activity may vary over time [36, 44]. The SHAP analysis confirmed that baseline BFI was the dominant predictor but did not materially alter the interpretation of the regression models. Clinically, this supports the need for repeated longitudinal fatigue assessment rather than relying solely on baseline profiling.

Taken together, these findings support conceptualizing fatigue in IA as a dynamic, state-dependent outcome rather than a stable trait measurable at a single time point. For clinical research and digital health monitoring, this argues for combining population-level estimates with individual-level trajectory analyses. These findings complement a companion analysis of this cohort that identified clinical–psychosocial archetypes predicting 12-week PtGADA outcomes [23]; the two studies address distinct research questions and employ different methodological frameworks, with the present study focusing specifically on fatigue trajectories, individual-level prediction, and extended longitudinal follow-up. A second companion analysis of the same underlying Mida Rheuma App cohort examined baseline predictors of 12-week pain improvement rather than fatigue [24]; together with the archetype study, these parallel analyses illustrate that different baseline symptom domains and self-management engagement patterns are associated with distinct 12-week outcomes in this population, reinforcing the rationale for domain-specific rather than one-size-fits-all digital self-management strategies. Beyond our own group’s work, other digital health studies published in this journal similarly support the feasibility and clinical relevance of app-based self-management in rheumatic disease: a pilot feasibility study of a different digital application in rheumatoid arthritis reported good usability and adherence over a comparably short observation window [45], and a German nationwide patient survey found substantial real-world uptake of prescribable digital health applications (DiGA) among people with rheumatic disease, alongside variable engagement over time [46]. The present study extends these predominantly short-term digital-health reports by providing individual-level longitudinal fatigue observations through 56 weeks.

### Strengths and limitations

The principal strengths of this study are the large baseline digital cohort, the real-world setting, and the explicit sensitivity analyses addressing differential loss to follow-up. The application of multiple MCID thresholds and complementary machine-learning prediction models adds analytical breadth.

Several limitations warrant acknowledgment. Follow-up rates were low (16.6% at W12, 2.5% at W52); the propensity model for W12 completion achieved only modest discrimination (AUC 0.624), and residual selection bias cannot be excluded despite IPW and sensitivity analyses. The action plan and medication analyses were exploratory and involved multiple nominal comparisons without adjustment for multiplicity; these findings should therefore be considered hypothesis-generating. The cohort was predominantly female and based mainly in German-speaking countries, limiting generalizability beyond digitally engaged populations. Baseline RAPID3 and PHQ-4 missingness required median imputation, and the ± 30-day landmark window may introduce temporal variability; these secondary analyses should therefore be interpreted as supportive rather than primary.

Several data-source constraints also limit interpretation. No objective inflammatory markers (C-reactive protein, erythrocyte sedimentation rate, joint counts) were collected, so this study characterises patient-reported fatigue patterns rather than objective disease activity; PtGADA, the only longitudinal disease-impact measure available, reflects subjective burden rather than inflammation. Longitudinal data on treatment changes and comorbidities were unavailable, so disease-related, therapeutic, and engagement-related explanations for the observed change in fatigue cannot be disentangled. Medication data, based on voluntary logging, covered only 23.7% of the cohort and likely overrepresented highly engaged users, precluding reliable adjustment for pharmacological confounding. Longitudinal changes in sleep, physical activity, and psychosocial status were not available in a form suitable for time-varying adjustment. Information on prior psychiatric diagnoses, psychiatric treatment history, and antidepressant or other psychotropic medication use was not collected.

Instrument-related caveats also apply. The BFI’s 1.0-point MCID is adapted from non-rheumatic contexts rather than formally validated for IA, and validated IA-specific fatigue instruments were not used. Similarly, the app-derived sleep-quality and social-support indices have not been independently validated against standard instruments.

## Conclusions

Fatigue trajectories in this real-world digital IA cohort were heterogeneous. In the primary longitudinal model, the average BFI change was small and attenuated after IPW adjustment, suggesting that apparent group-level improvement was sensitive to differential loss to follow-up. In secondary landmark analyses, approximately one quarter of observed patients experienced clinically meaningful BFI worsening at each assessed time point. Baseline BFI was the dominant predictor of both W12 BFI improvement and W12 BFI worsening, consistent with regression to the mean. Engagement with selected digital self-management action plan categories, particularly Diet & nutrition and Disease education, was associated with more favorable W12 BFI outcomes in exploratory analyses; higher total task completion was associated with lower odds of W12 BFI worsening. However, causal inference is not supported by the current design. These findings support further prospective evaluation of longitudinal, individual-level fatigue monitoring and targeted digital self-management strategies in IA.

## Supplementary Information

Below is the link to the electronic supplementary material.

Supplementary Material 1

Supplementary Material 2

## Data Availability

Analysis was conducted on anonymized data, under a data-processing agreement, which allowed the authors to maintain independent analytic control. Data sharing: Midaia GmbH data is not approved for sharing.

## Declarations

### Open Access Publishing note

The corresponding author, Stefan Vordenbäumen, meets the reimbursement requirements of Heinrich Heine University Düsseldorf, which has an agreement with Springer Nature that can cover article processing charges.
