# Supplementary material for: Longitudinal fatigue outcomes and prediction in inflammatory arthritis: a real-world digital cohort study
Source: Rheumatol Int. 2026 Sep 30;46(10):270. doi: 10.1007/s00296-026-06308-9 (PMC13630824; doi:10.1007/s00296-026-06308-9)
Supplement: Supplementary file 1 [file 296_2026_6308_MOESM1_ESM.docx]

# STROBE Statement — Checklist of Items that Should Be Included in Reports of Observational Studies

*Manuscript: "Longitudinal fatigue outcomes and prediction in inflammatory arthritis: a real-world digital cohort study" (cohort study).*

| **Item No** | **Section** | **Recommendation** | **Reported on manuscript page/section** |
| --- | --- | --- | --- |
| 1a | Title and abstract | Indicate the study's design with a commonly used term in the title or the abstract. | Title ("...a real-world digital cohort study") identifies the study design via the term “cohort study”; the retrospective design is specified in Methods – Study Design and Participants, first sentence. |
| 1b | Title and abstract | Provide in the abstract an informative and balanced summary of what was done and what was found. | Abstract (structured: Background, Objective, Methods, Results, Conclusion). |
| 2 | Introduction — Background/rationale | Explain the scientific background and rationale for the investigation being reported. | Introduction, opening paragraphs (fatigue burden in inflammatory arthritis, gaps in longitudinal digital-cohort evidence, rationale for the BFI instrument). |
| 3 | Introduction — Objectives | State specific objectives, including any prespecified hypotheses. | Introduction, final paragraph (five numbered study aims). |
| 4 | Methods — Study design | Present key elements of study design early in the paper. | Methods – Study Design and Participants, first paragraph (retrospective real-world cohort study; STROBE-guided reporting stated explicitly). |
| 5 | Methods — Setting | Describe the setting, locations, and relevant dates, including periods of recruitment, exposure, follow-up, and data collection. | Methods – Study Design and Participants (Mida Rheuma App, Midaia GmbH, Germany; data collected January 2022–March 2026); the predominantly German-speaking user base is additionally noted in the Introduction. |
| 6a | Methods — Participants | Cohort study: give the eligibility criteria, and the sources and methods of selection of participants. Describe methods of follow-up. | Methods – Study Design and Participants (eligibility criteria: age ≥18 years, consent for research use, eligible IA diagnosis, ≥1 valid baseline BFI); follow-up timing and window methodology are described in App Assessments and Follow-up Structure; and Figure 1 (STROBE participant-flow diagram). |
| 6b | Methods — Participants | For matched studies, give matching criteria and number of exposed/unexposed. | Not applicable — unmatched cohort study. |
| 7 | Methods — Variables | Clearly define all outcomes, exposures, predictors, potential confounders, and effect modifiers. Give diagnostic criteria, if applicable. | Methods – Clinical Variables and Outcomes (BFI outcome and MCID definition) and baseline covariate definitions (PPAIN, PtGADA, RAPID3, PHQ-4 with depression/anxiety subscales, sleep-quality and social-support composite indices, medication categories). |
| 8 | Methods — Data sources/measurement | For each variable of interest, give sources of data and details of methods of assessment. Describe the comparability of assessment methods if there is more than one group. | Methods – App Assessments and Follow-up Structure and Clinical Variables and Outcomes (all variables collected through the single Mida Rheuma App platform; assessment timing and instrument sources specified per variable). |
| 9 | Methods — Bias | Describe any efforts to address potential sources of bias. | Methods – Statistical Analysis (comparison of W12 completers and non-completers; inverse probability weighting based on measured baseline predictors of follow-up completion; E-values for sensitivity of baseline-predictor associations to unmeasured confounding; tipping-point analysis for residual bias in the longitudinal slope); Discussion – Strengths and Limitations. |
| 10 | Methods — Study size | Explain how the study size was arrived at. | Methods – Study Design and Participants: no a priori sample-size calculation was performed; all eligible users meeting the prespecified inclusion criteria during the study period were included. Figure 1 shows the derivation of the analytic cohort from 4,915 registered users to 2,660 participants. |
| 11 | Methods — Quantitative variables | Explain how quantitative variables were handled in the analyses. If applicable, describe which groupings were chosen and why. | BFI modelled continuously via linear regression on time since baseline; secondary landmark analyses used a prespecified pragmatic 1.0-point BFI change threshold, with alternative thresholds examined in sensitivity analyses. |
| 12a | Methods — Statistical methods | Describe all statistical methods, including those used to control for confounding. | Methods – Statistical Analysis, full subsection (cluster-robust linear regression, multivariable logistic regression, elastic net and gradient boosting with SHAP interpretation, and inverse probability weighting); adjustment covariates were selected a priori based on clinical relevance to fatigue outcomes and availability within the app dataset. |
| 12b | Methods — Statistical methods | Describe any methods used to examine subgroups and interactions. | Not applicable — no prespecified subgroup or statistical interaction analyses were performed. Diagnosis category was included as a covariate in the multivariable models; medication-class comparisons were conducted as descriptive, exploratory analyses. |
| 12c | Methods — Statistical methods | Explain how missing data were addressed. | Methods – Statistical Analysis, missing-data paragraph: BMI, RAPID3, and PHQ-4 were median-imputed in multivariable models; imputation was performed within the cross-validation pipeline for machine-learning analyses; follow-up BFI values were not imputed. |
| 12d | Methods — Statistical methods | Cohort study: if applicable, explain how loss to follow-up was addressed. | Methods – Statistical Analysis, loss-to-follow-up paragraph (propensity model for W12 completion; inverse probability weighting). |
| 12e | Methods — Statistical methods | Describe any sensitivity analyses. | Methods – Statistical Analysis: alternative BFI change thresholds, IPW-adjusted analyses, E-values for unmeasured confounding of baseline-predictor associations, tipping-point analysis for the longitudinal slope, and an exploratory W24 machine-learning analysis. |
| 13a | Results — Participants | Report numbers of individuals at each stage of study. | Figure 1 (STROBE participant-flow diagram, numbers at each eligibility/inclusion stage) and Results – Cohort Characteristics, opening paragraph. |
| 13b | Results — Participants | Give reasons for non-participation at each stage. | Figure 1 legend and Methods – Study Design and Participants (exclusion reasons: age, consent, diagnosis, missing baseline BFI). |
| 13c | Results — Participants | Consider use of a flow diagram. | Figure 1 (STROBE participant-flow diagram). |
| 14a | Results — Descriptive data | Give characteristics of study participants and information on exposures and potential confounders. | Results – Cohort Characteristics and Table 1 (demographics, diagnosis distribution, baseline PRO scores); medication-class distribution is reported separately in Results text (Cohort Characteristics), not in Table 1. |
| 14b | Results — Descriptive data | Indicate number of participants with missing data for each variable of interest. | Results – Cohort Characteristics (denominators reported per variable in text) and Supplementary Table S1 (missingness counts and percentages per variable). |
| 14c | Results — Descriptive data | Cohort study: summarise follow-up time. | Results – Longitudinal Fatigue Trajectories and Loss to Follow-up and IPW subsections (follow-up availability at W12, W24, and W52 reported). |
| 15 | Results — Outcome data | Cohort study: report numbers of outcome events or summary measures over time. | Results – Fatigue Improvement and Worsening Analysis (counts and proportions of clinically meaningful BFI improvement/worsening at each landmark time point). |
| 16a | Results — Main results | Give unadjusted estimates and, if applicable, confounder-adjusted estimates and their precision. Make clear which confounders were adjusted for and why. | Results – Longitudinal Fatigue Trajectories and Loss to Follow-up/IPW (unadjusted and IPW-adjusted estimates with 95% CIs) and Predictors of W12 BFI Improvement and Worsening (adjusted odds ratios with 95% CIs); Methods states that adjustment covariates were selected a priori based on clinical relevance and data availability. |
| 16b | Results — Main results | Report category boundaries when continuous variables were categorized. | Prespecified pragmatic ±1.0-point BFI change threshold for improvement/worsening, with alternative thresholds specified for sensitivity analyses; PHQ-4 subscale cut-off ≥3 stated explicitly. |
| 16c | Results — Main results | If relevant, consider translating estimates of relative risk into absolute risk for a meaningful time period. | Results – Longitudinal Fatigue Trajectories (unadjusted weekly slope translated into an approximate absolute BFI-point change by week 12). |
| 17 | Results — Other analyses | Report other analyses done — eg analyses of subgroups and interactions, and sensitivity analyses. | Results – Self-Management Action Plan Engagement, Machine-Learning Prediction of W12 BFI Outcomes, Loss to Follow-up/IPW, and descriptive baseline medication-class analyses; exploratory W24 machine-learning results and alternative-threshold analyses are reported in the Supplement. |
| 18 | Discussion — Key results | Summarise key results with reference to study objectives. | Discussion, opening paragraph. |
| 19 | Discussion — Limitations | Discuss limitations of the study, taking into account sources of potential bias or imprecision. Discuss both direction and magnitude of any potential bias. | Discussion – Strengths and Limitations: low and differential follow-up, residual selection bias, multiplicity of exploratory analyses, medication-data coverage, missing time-varying clinical/psychosocial factors, absence of objective inflammatory markers, and instrument-related limitations are explicitly discussed. |
| 20 | Discussion — Interpretation | Give a cautious overall interpretation of results considering objectives, limitations, multiplicity of analyses, results from similar studies, and other relevant evidence. | Discussion, middle paragraphs (regression to the mean, self-management engagement signal, comparison with prior literature and with the group's companion studies). |
| 21 | Discussion — Generalisability | Discuss the generalisability (external validity) of the study results. | Discussion – Strengths and Limitations: the predominantly female, mainly German-speaking, digitally engaged cohort is acknowledged, and the limited generalizability beyond digitally engaged populations is explicitly discussed. |
| 22 | Other information — Funding | Give the source of funding and the role of the funders for the present study. | Funding/Role of Sponsor declaration (Title Page / Declarations section): EULAR Research Grant and Midaia GmbH data-infrastructure support, with an explicit statement of no funder role in study design, analysis, interpretation, or the decision to submit. |

**Give information separately for exposed and unexposed groups where applicable. An Explanation and Elaboration article discusses each checklist item (www.strobe-statement.org).*
