# Supplementary material for: Longitudinal fatigue outcomes and prediction in inflammatory arthritis: a real-world digital cohort study
Source: Rheumatol Int. 2026 Sep 30;46(10):270. doi: 10.1007/s00296-026-06308-9 (PMC13630824; doi:10.1007/s00296-026-06308-9)
Supplement: Supplementary file 2 [file 296_2026_6308_MOESM2_ESM.docx]

**SUPPLEMENTARY MATERIAL**

**Supplementary Table S1. Missing Baseline Data for Candidate Predictors**

| **Variable** | **N available (%)** | **N missing (%)** | **Handling in analysis** |
| --- | --- | --- | --- |
| BFI (baseline) | 2,660 (100%) | 0 (0%) | Primary outcome; complete by inclusion criterion |
| PPAIN (baseline) | 2,660 (100%) | 0 (0%) | Complete |
| PtGADA (baseline) | 2,660 (100%) | 0 (0%) | Complete |
| Age | 2,660 (100%) | 0 (0%) | Complete |
| Sex | 2,660 (100%) | 0 (0%) | Complete |
| BMI (baseline) | 2,468 (92.8%) | 192 (7.2%) | Median imputation in multivariable models |
| RAPID3 (baseline) | 2,056 (77.3%) | 604 (22.7%) | Median imputation in multivariable models |
| PHQ-4 (baseline) | 2,061 (77.5%) | 599 (22.5%) | Median imputation in multivariable models |
| Sleep Quality Score (baseline) | 2,519 (94.7%) | 141 (5.3%) | Descriptive characterization only |
| Social Support Score (baseline) | 2,466 (92.7%) | 194 (7.3%) | Descriptive characterization only |

Notes: Availability is shown for the primary analytic cohort. Abbreviations: BFI, Brief Fatigue Inventory; BMI, body mass index; PHQ-4, Patient Health Questionnaire-4; PPAIN, patient-reported pain score; PtGADA, Patient Global Assessment of Disease Activity; RAPID3, Routine Assessment of Patient Index Data 3.

**Supplementary Table S2. Baseline Characteristics: W12 Completers vs Non-Completers**

| **Variable** | **W12 completers (N=441)** | **W12 non-completers (N=2,219)** | **SMD** | **p-value** |
| --- | --- | --- | --- | --- |
| BFI baseline | 4.8 (±2.0) | 5.3 (±1.9) | 0.26 | <0.001 |
| PPAIN baseline | 49.8 (±25.2) | 54.6 (±25.6) | 0.19 | <0.001 |
| PtGADA baseline | 54.5 (±24.2) | 59.3 (±25.6) | 0.19 | <0.001 |
| RAPID3 baseline | 11.9 (±5.2) | 13.0 (±5.3) | 0.21 | <0.001 |
| PHQ-4 baseline | 3.8 (±2.5) | 4.3 (±2.8) | 0.19 | 0.001 |
| Age (years) | 49.4 (±12.6) | 44.8 (±12.8) | 0.36 | <0.001 |
| Female sex, n (%) | 352 (79.8%) | 1,731 (78.0%) | 0.04 | 0.399 |
| Sleep Quality Score (baseline) | 0.59 (±1.74) | 0.37 (±1.72) | 0.13 | 0.017 |
| Social Support Score (baseline) | 13.70 (±4.30) | 13.20 (±4.27) | 0.12 | 0.029 |

Notes: Values are mean (SD) unless otherwise stated. P-values are descriptive and should be interpreted alongside SMDs. SMD values >0.10 indicate a potentially meaningful imbalance. Abbreviations: BFI, Brief Fatigue Inventory; BMI, body mass index; IPW, inverse probability weighting; PHQ-4, Patient Health Questionnaire-4; PPAIN, patient-reported pain score; PtGADA, Patient Global Assessment of Disease Activity; RAPID3, Routine Assessment of Patient Index Data 3; SD, standard deviation; SMD, standardized mean difference; W12, week 12.

**Supplementary Table S3. Clinically meaningful BFI improvement and worsening under alternative MCID thresholds**

| **Timepoint** | **MCID threshold** | **N** | **BFI improvement n (%)** | **Stable n (%)** | **BFI worsening n (%)** |
| --- | --- | --- | --- | --- | --- |
| W12 | Primary (1.0) | 441 | 118 (26.8%) | 210 (47.6%) | 113 (25.6%) |
| W12 | Sensitivity A (±1.33) | 441 | 85 (19.3%) | 264 (59.9%) | 92 (20.9%) |
| W12 | Sensitivity B (-1.5/1.7) | 441 | 73 (16.6%) | 308 (69.8%) | 60 (13.6%) |
| W24 | Primary (1.0) | 259 | 74 (28.6%) | 116 (44.8%) | 69 (26.6%) |
| W24 | Sensitivity A (±1.33) | 259 | 61 (23.6%) | 147 (56.8%) | 51 (19.7%) |
| W24 | Sensitivity B (-1.5/1.7) | 259 | 54 (20.8%) | 168 (64.9%) | 37 (14.3%) |
| W52 | Primary (1.0) | 67 | 22 (32.8%) | 28 (41.8%) | 17 (25.4%) |
| W52 | Sensitivity A (±1.33) | 67 | 18 (26.9%) | 39 (58.2%) | 10 (14.9%) |
| W52 | Sensitivity B (-1.5/1.7) | 67 | 17 (25.4%) | 43 (64.2%) | 7 (10.4%) |

Notes: ΔBFI was calculated as follow-up BFI minus baseline BFI. Sensitivity A used symmetric thresholds of ±1.33 BFI points. Sensitivity B used an improvement threshold of −1.5 and a worsening threshold of +1.7 BFI points. Abbreviations: BFI, Brief Fatigue Inventory; MCID, minimal clinically important difference; W12/W24/W52, week 12/week 24/week 52.

**Supplementary Table S4. Machine learning prediction performance**

| **Outcome** | **Model** | **AUC (mean** ± **SD)** | **AUPRC (mean** ± **SD)** | **N** | **Events** |
| --- | --- | --- | --- | --- | --- |
| W12 BFI improvement | Elastic Net | 0.651 ± 0.048 | 0.398 ± 0.062 | 441 | 118 |
| W12 BFI improvement | Gradient Boosting | 0.603 ± 0.057 | 0.387 ± 0.066 | 441 | 118 |
| W12 BFI worsening | Elastic Net | 0.720 ± 0.066 | 0.490 ± 0.063 | 441 | 113 |
| W12 BFI worsening | Gradient Boosting | 0.680 ± 0.065 | 0.449 ± 0.067 | 441 | 113 |
| W24 BFI improvement | Elastic Net | 0.669 ± 0.060 | 0.444 ± 0.044 | 259 | 74 |
| W24 BFI improvement | Gradient Boosting | 0.711 ± 0.050 | 0.579 ± 0.074 | 259 | 74 |

Notes: Values are mean ± SD across five stratified cross-validation folds. Preprocessing, including median imputation and standardization, was performed within the cross-validation pipeline. Events denote the number of patients with BFI improvement or BFI worsening, according to the corresponding outcome definition. W24 BFI improvement performance is shown as an exploratory supplementary analysis. Abbreviations: AUC, area under the receiver operating characteristic curve; AUPRC, area under the precision-recall curve; BFI, Brief Fatigue Inventory; SD, standard deviation; W12/W24, week 12/week 24.

**Supplementary Table S5. Sensitivity of action plan associations to MCID threshold definition (W12, adjusted models)**

| **MCID threshold** | **Action plan category** | **OR for W12 improvement (95% CI; p)** | **OR for W12 worsening (95% CI; p)** |
| --- | --- | --- | --- |
| Primary (±1.0) | Diet & nutrition | 1.78* (1.07–2.96; p=0.026) | 0.45* (0.27–0.75; p=0.002) |
|  | Disease education | 1.91* (1.16–3.16; p=0.011) | 0.63 (0.39–1.02; p=0.063) |
|  | Exercise & movement | 1.19 (0.76–1.86; p=0.457) | 0.75 (0.46–1.21; p=0.240) |
|  | Total tasks (per 10) | 1.02 (0.98–1.05; p=0.379) | 0.95* (0.92–1.00; p=0.030) |
| Sens A (±1.33) | Diet & nutrition | 1.52 (0.87–2.64; p=0.142) | 0.40* (0.23–0.69; p=0.001) |
|  | Disease education | 1.46 (0.85–2.51; p=0.172) | 0.53* (0.31–0.90; p=0.019) |
|  | Exercise & movement | 1.05 (0.64–1.73; p=0.839) | 0.69 (0.41–1.16; p=0.158) |
|  | Total tasks (per 10) | 1.00 (0.96–1.04; p=0.924) | 0.95* (0.90–0.99; p=0.021) |
| Sens B (−1.5/+1.7) | Diet & nutrition | 1.89* (1.03–3.47; p=0.041) | 0.55 (0.28–1.07; p=0.078) |
|  | Disease education | 1.50 (0.84–2.68; p=0.166) | 0.47* (0.25–0.90; p=0.023) |
|  | Exercise & movement | 1.03 (0.61–1.74; p=0.917) | 0.88 (0.47–1.67; p=0.703) |
|  | Total tasks (per 10) | 0.99 (0.95–1.03; p=0.628) | 0.95 (0.90–1.01; p=0.081) |

Notes: Odds ratios from adjusted logistic regression models for W12 BFI improvement vs no improvement, and W12 BFI worsening vs no worsening. Adjusted covariates: baseline BFI, PPAIN, PtGADA, PHQ-4, RAPID3, age, and sex. The primary threshold (±1.0) is the main analysis threshold. Sensitivity A used symmetric thresholds of ±1.33 BFI points. Sensitivity B used asymmetric thresholds of −1.5 for improvement and +1.7 for worsening. *p<0.05. Abbreviations: BFI, Brief Fatigue Inventory; CI, confidence interval; MCID, minimal clinically important difference; OR, odds ratio; PPAIN, patient-reported pain score; PtGADA, Patient Global Assessment of Disease Activity; W12, week 12.

**Supplementary Table S6. Logistic Regression Model for W12 Follow-up Completion**

| **Predictor** | **OR** | **95% CI** | **p-value** |
| --- | --- | --- | --- |
| BFI (baseline) | 0.945 | 0.879 – 1.017 | 0.129 |
| PPAIN (baseline) | 0.999 | 0.992 – 1.006 | 0.787 |
| PtGADA (baseline) | 0.998 | 0.991 – 1.006 | 0.666 |
| RAPID3 (baseline) | 0.990 | 0.956 – 1.025 | 0.563 |
| PHQ-4 (baseline) | 0.984 | 0.933 – 1.038 | 0.547 |
| Age (per year) | 1.028 | 1.019 – 1.036 | <0.001 |
| Male sex | 1.369 | 1.049 – 1.787 | 0.021 |
| BMI (baseline) | 0.987 | 0.971 – 1.004 | 0.142 |

Notes: The dependent variable was the availability of the W12 BFI follow-up. Odds ratios >1 indicate higher odds of W12 completion. This model was used to estimate stabilized inverse probability weights. Model AUC was 0.624. Abbreviations: AUC, area under the receiver operating characteristic curve; BFI, Brief Fatigue Inventory; BMI, body mass index; CI, confidence interval; OR, odds ratio; PHQ-4, Patient Health Questionnaire-4; PPAIN, patient-reported pain score; PtGADA, Patient Global Assessment of Disease Activity; RAPID3, Routine Assessment of Patient Index Data 3; W12, week 12.

**Figure S1. Spearman correlation heatmap of baseline clinical and psychological variables.** Cells show Spearman’s rho correlation coefficients between baseline variables. Abbreviations: BFI, Brief Fatigue Inventory; PHQ-4, Patient Health Questionnaire-4; PPAIN, patient-reported pain score; PtGADA, Patient Global Assessment of Disease Activity; RAPID3, Routine Assessment of Patient Index Data 3.

**Figure S2. Covariate balance before and after inverse probability weighting.** The Love plot shows absolute standardized mean differences for baseline covariates comparing W12 completers and non-completers before and after IPW. Reference lines indicate SMD thresholds of 0.10 and 0.20. Abbreviations: BFI, Brief Fatigue Inventory; BMI, body mass index; IPW, inverse probability weighting; PHQ-4, Patient Health Questionnaire-4; PPAIN, patient-reported pain score; PtGADA, Patient Global Assessment of Disease Activity; RAPID3, Routine Assessment of Patient Index Data 3; SMD, standardized mean difference; W12, week 12.

**Figure S3. Distribution of baseline BFI scores across W12 fatigue outcome groups.** The plot combines density distributions, box-and-whisker summaries, and individual data points. It illustrates the regression-to-the-mean pattern in secondary time-point analyses: patients classified as having BFI improvement tended to have higher baseline fatigue, whereas patients classified as having BFI worsening tended to have lower baseline fatigue. Abbreviations: BFI, Brief Fatigue Inventory; W12, week 12.

**Figure S4. Discrimination and calibration of W12 BFI improvement prediction models.** Panel A shows receiver operating characteristic curves and Panel B shows calibration using decile-binned out-of-fold predictions from five-fold cross-validation. The diagonal line represents perfect calibration. Abbreviations: AUC, area under the receiver operating characteristic curve; ROC, receiver operating characteristic; W12, week 12.

**Figure S5. SHAP summary plot for the gradient boosting model predicting W12 BFI improvement.** Predictors are ranked by mean absolute SHAP value. Each point represents one patient; the position on the x-axis indicates the feature's contribution to the model output, and the color indicates the observed feature value. The SHAP analysis was used as a complementary non-linear interpretability analysis. Abbreviations: BFI, Brief Fatigue Inventory; BMI, body mass index; PHQ-4, Patient Health Questionnaire-4; PPAIN, patient-reported pain score; PtGADA, Patient Global Assessment of Disease Activity; RAPID3, Routine Assessment of Patient Index Data 3; SHAP, SHapley Additive exPlanations; W12, week 12.

**Figure S1**

**Figure S2**

**Figure S3**

**Figure S4**

**Figure S5**
